# Zoonotic and other veterinary chlamydiae – an update, the role of the plasmid and plasmid-mediated transformation

**DOI:** 10.1093/femspd/ftae030

**Published:** 2024-11-20

**Authors:** Hanna Marti, Kensuke Shima, Sebastien Boutin, Jan Rupp, Ian N Clarke, Karine Laroucau, Nicole Borel

**Affiliations:** Institute of Veterinary Pathology, University of Zurich, 8057 Zurich, Switzerland; Institute of Medical Microbiology, University of Lübeck, 23538 Lübeck, Germany; Institute of Medical Microbiology, University of Lübeck, 23538 Lübeck, Germany; Airway Research Center North (ARCN), German Center for Lung Research (DZL), Lübeck, Germany; Institute of Medical Microbiology, University of Lübeck, 23538 Lübeck, Germany; German Center for Infection Research, Partner Site Hamburg-Lübeck-Borestel-Riems, Lübeck, Germany; Clinic for Infectious Diseases, University of Lübeck, 23538, Germany; Molecular Microbiology, School of Clinical and Experimental Sciences, School of Medicine, University of Southampton, SO17 1BJ Southampton, United Kingdom; University Paris-Est, ANSES, Animal Health Laboratory, Bacterial Zoonoses Unit, 94700 Maisons-Alfort, France; Institute of Veterinary Pathology, University of Zurich, 8057 Zurich, Switzerland

**Keywords:** *Chlamydia*, zoonotic, transformation, plasmid, veterinary

## Abstract

The obligate intracellular bacterial genus *Chlamydia* harbours species with zoonotic potential, particularly *C. psittaci*, causative agent of psittacosis, and *C. abortus*, which may lead to miscarriage in pregnant women. The impact of other bird chlamydiae such as *C. avium, C. gallinaceae*, and *C. buteonis*, or reptilian species such as *C. crocodili*, amongst others, on human health is unclear. The chlamydial native plasmid, a suspected virulence factor, is present in all currently described 14 *Chlamydia* species except for some plasmid-free strains. The plasmid is also the primary tool to study chlamydial genetics, a still developing field that has mostly focused on *C. trachomatis*. Only recently, genetic transformation of *C. felis, C. pecorum, C. pneumoniae, C. psittaci*, and *C. suis* has succeeded, but existing methods have yet to be refined. In this review article, we will provide an update on the recent developments concerning the zoonotic potential of chlamydiae. Furthermore, we present an overview about the current state of knowledge regarding the chlamydial plasmid in terms of prevalence and significance as a virulence factor. Finally, we give insights into the progress of developing genetic tools for chlamydial species other than *C. trachomatis* with a special focus on zoonotic and veterinary chlamydiae.

## Teaser

This article focuses on an update on zoonotic and other veterinary chlamydiae and their genetic manipulation.

## Update on zoonotic and nonzoonotic veterinary chlamydiae

The bacterial family *Chlamydiaceae* currently comprises the genus *Chlamydia* with 14 officially accepted species (Table 1) and a very recently added genus *Chlamydiifrater* with two new species, *Chlamydiifrater phoenicopteri* sp. nov. and *Chlamydiifrater volucris* sp. nov., both isolated from wild flamingos (*Phoenicopterus roseus*) (Vorimore et al. 2021). Of all the currently accepted *Chlamydiaceae* species (Luu et al. 2023), the most studied species is *C. trachomatis*, restricted to human hosts and responsible for a chronic eye infection, termed trachoma, as well as the most common cause of bacterial sexually transmitted infections (STI) worldwide (Jordan et al. 2020). Of the remaining species, four nonhuman chlamydiae possess a confirmed zoonotic potential, namely *C. psittaci, C. abortus, C. caviae*, and *C. felis*, which have been extensively reviewed (Cheong et al. 2019, Sachse and Borel 2020, Borel and Sachse 2022). The focus of this chapter is to provide a brief update on well-known zoonotic chlamydial species and to further explore animal-to-human transmission of newly discovered chlamydial species in birds, livestock, pets, and exotic animals, as well as to look into uncommon reservoirs of zoonotic infection.

**Table 1.** Family *Chlamydiaceae*, genus *Chlamydia*, 14 species with main hosts, zoonotic potential, and currently available genetic tools.

Chlamydial species	Main animal host(s)	Zoonotic	Plasmid-free strains	Genetic tools	References pertaining genetic modification
*C. psittaci*	Birds, horses	Yes	Yes (Sachse et al. 2023)	Plasmid shuttle vector, allelic exchange vector	Binet and Maurelli (2009), Shima et al. (2020)
*C. abortus*	Ruminants (mammalian), birds (avian)	Yes	All ruminant strains	–	–
*C. caviae*	Guinea pigs	Yes	No reports	Plasmid shuttle vector, TargeTron	Filcek et al. (2019), Faessler et al. (2024)
*C. felis* ^a^	Cats	Yes	Yes	Plasmid shuttle vector	Shima et al. (2018)
*C. suis*	Pigs	(Yes)	Yes	Plasmid shuttle vector, allelic exchange vector	Marti et al. (2023)
*C. pecorum*	Ruminants, pigs, koala	(Yes)	Yes	Plasmid shuttle vector	Faessler et al. (2024)
*C. gallinacea*	Birds, (cattle)	(Yes)	No reports	–	–
*C. buteonis*	Birds	No	No reports	–	–
*C. avium*	Birds	No	No reports	–	–
*C. poikilotherma*	Snakes	No	No reports	–	–
*C. trachomatis*	Humans	No	Yes	Plasmid shuttle vector, FRAEM, FLAEM, CRISPRi, sRNAs, transposon mutagenesis, TargeTron	Reviewed in: Bastidas and Valdivia (2016), Sixt and Valdivia (2016), Andersen et al. (2021), Wan et al. (2023)
*C. pneumoniae*	Humans, reptiles, horses, koalas	No	Yes (all human isolates)	Plasmid shuttle vector	Gérard et al. (2013), Shima et al. (2018)
*C. muridarum*	Rodents	No	Curation possible	Plasmid shuttle vector, transposon mutagenesis	Wang et al. (2013, 2019), Song et al. (2014), Skilton et al. (2018)
*C. crocodili*	Crocodiles	No	No (few isolates available)	–	–

a *Chlamydia felis* was successfully transformed with a *C. pneumoniae* but not *C. felis-*specific plasmid shuttle vector (Shima et al. 2018).

### *Chlamydia* sp. in birds

Domestic and wild birds are among the most common sources for zoonotic chlamydial infections, for which, until recently, *C. psittaci* was considered to be the primary infecting chlamydial species (recently reviewed in Ravichandran et al. 2021). Historical epidemics of psittacosis were documented from the late nineteenth century to the 1930s, often associated with the trade of exotic birds. Since then, *C. psittaci* has been detected in many different avian hosts, including pigeons, poultry, and wild birds, often following human cases. However, recent research has identified new species of *Chlamydia* in birds, with or without clinical signs in their hosts. These discoveries have broadened the definition of ‘avian chlamydiosis’ to include *C. gallinacea, C. avium, C. buteonis*, and *Candidatus* C. ibidis.

*C. psittaci* is probably the most important species of the veterinary chlamydiae from a One Health perspective. It has been described in over 460 species of wild and captive birds worldwide (Kaleta and Taday 2003), although nonspecific serological testing in the past may have overestimated its presence while missing the more recently described species. Based on genomic analyses, *C. psittaci* is divided into two genotypes (WC and M56) isolated from mammals and six avian genotypes identified in psittacines, ducks, pigeons, and other birds (Sachse et al. 2023). All avian genotypes of *C. psittaci* have a zoonotic potential, with the psittacine strains most commonly implicated in human infections, particularly. Community-acquired pneumonia (CAP), to which *C. psittaci* contributes 1% of all cases (Hogerwerf et al. 2017). Human psittacosis/ornithosis presents with mild to severe respiratory symptoms, including fever, pneumonia, myocarditis, encephalitis, and splenomegaly, and may require hospitalization (Dembek et al. 2023). Zoonotic infection usually occurs through inhalation of feather dust or dried faeces after contact with an infected animal. High-risk groups include breeders, veterinarians, pet shop, or bird care centre staff as well as slaughterhouse workers. Veterinary laboratory personnel handling infectious material are also at risk of infection. Acute clinical signs predominate in young birds, while infections often remain mild or asymptomatic in older birds. Clinical signs in birds include coughing, respiratory distress, conjunctivitis, nasal and ocular discharge, and sometimes greenish diarrhoea (Hogerwerf et al. 2020). Disease outbreaks in certain geographical regions can cause significant economic losses to the poultry industry due to carcass condemnation, reduced egg production, mortality, and antibiotic treatment costs, as well as posing an ongoing zoonotic risk. Interestingly, the host specificity of *C. psittaci* is not strict, and strains have been isolated from nonavian species such as dogs (Sprague et al. 2009) and, more recently, from horses in Australia (Anstey et al. 2021). Zoonotic transmission to human from equine abortion cases caused by *C. psittaci* has been described (Chan et al. 2017).

Over the last 20 years, several strains have been isolated from birds that were initially described as ‘similar to *C. psittaci*’ or ‘atypical’, before they were revisited and better characterized. This has led to the description of new species. *C. gallinacea* and *C. avium* were among the first new species described, marking the expansion of the *Chlamydiaceae* family in birds (Sachse et al. 2014). In 2009, an investigation in a poultry slaughterhouse in France revealed atypical chlamydiae isolated from chickens, which were distinct from *C. psittaci* (Laroucau et al. 2009). Phylogenetic analysis and whole genome sequencing later led to the description of *C. gallinacea* in 2014 (Sachse et al. 2014). Although these strains were initially suspected of causing human pneumonia cases, this has never been confirmed. *C. gallinacea* is primarily found in chickens, although its presence has anecdotally been demonstrated in other birds such as pigeons, woodcock, and parrots, as well as in ruminants (Guo et al. 2016, Li et al. 2016, Stokes et al. 2019). Globally distributed, genome analysis of *C. gallinacea* reveals significant heterogeneity within the species, although it is not yet known whether these genetic differences have functional implications for host adaptation or strain virulence (Heijne et al. 2021). Additionally, a retrospective study of chlamydial strains isolated from pigeons in Italy and France, and from tissues collected from psittacines with clinical signs in Germany, led to the description of *C. avium* in 2014 (Sachse et al. 2014). *C. avium* is primarily detected in pigeons and psittaciform birds, although its presence in other birds is suspected and anecdotally demonstrated.

*C. buteonis*, a chlamydial species closely related to *C. psittaci* and *C. abortus*, and so far exclusively isolated from birds of prey, can cause clinical signs such as conjunctivitis and may be fatal in extreme cases, although an asymptomatic carrier status appears to predominate. *C. buteonis* has been identified in birds of prey both in the USA (Mirandé et al. 1992, Laroucau et al. 2019) and more recently in the United Arab Emirates (UAE) (Stalder et al. 2021), with different genotypes for which it is not known whether they are related to geographical regions (USA versus UAE) or hosts (falcon versus hawk) (Vorimore et al. 2021).

Finally, during sampling of wild ibises in France to assess their potential role in transmission of *C. psittaci* to ducks, atypical chlamydial strains were isolated, which were then identified as *Candidatus* C. ibidis (Vorimore et al. 2013). This *Candidatus* species has been isolated from healthy African sacred ibises in France and more recently from crested ibises in China (Li et al. 2020) and wild birds in Australia (Kasimov et al. 2022), indicating worldwide distribution.

It is likely that other avian species will continue to enrich this expanding family. As *C. psittaci* is the only species with confirmed zoonotic potential, the terms ‘psittacosis’ or ‘ornithosis’ are still used to describe *C. psittaci* infections in humans (Borel and Greub 2021).

### *Chlamydia* sp. in livestock

*Chlamydia abortus* is the most common infectious abortigenic agent in sheep and goats in Europe and, to a lesser extent, in other wild and domestic ruminants, pigs, and horses (Buxton 1986, Hyde and Benirschke 1997, Longbottom and Coulter 2003, Borel et al. 2018). This pathogen is well known to pose a significant risk to pregnant women leading to miscarriage (Pospischil et al. 2002, Essig and Longbottom 2015, Burgener et al. 2022). Recent uncommon clinical presentations in pregnant women included the development of acute respiratory distress syndrome (Pichon et al. 2020) and severe atypical pneumonia (Imkamp et al. 2022). All historical and recent cases have been linked to direct or indirect contact to aborting or lambing sheep and goats but not to other livestock. A 65-year-old male patient suffering of septic shock due to *C. abortus* denied any animal contact (Liu et al. 2022), highlighting the need to explore sources of environmental exposure to this and other chlamydial species (Turin et al. 2022). Novel *C. abortus* strains have recently been found in birds, initially interpreted as intermediates of *C. abortus* and *C. psittaci* (Szymańska-Czerwińska et al. 2017, 2023, Origlia et al. 2019, Zaręba-Marchewka et al. 2020, 2021, Longbottom et al. 2021, Stokes et al. 2021, Aaziz et al. 2023, Kasimov et al. 2023). These novel *C. abortus* strains harbour a plasmid (Zaręba-Marchewka et al. 2021) and may cause pneumonia in humans (Raven et al. 2024).

Another common livestock pathogen, *C. pecorum*, known to cause abortion, encephalomyelitis, and polyarthritis in ruminants, has so far not been considered a zoonotic pathogen (Sachse and Borel 2020). There is one single case report of a sheep farmer suffering from severe CAP. *C. pecorum* was detected in the broncho-alveolar lavage of this patient, indicating some level of zoonotic transmission (Cao et al. 2022). Although no causative relationship between CAP and *C. pecorum* infection could be demonstrated, the assumption that *C. pecorum* is not zoonotic should undergo some reassessment.

*C. suis*, highly adapted to and endemic in pigs, mostly colonizes the intestinal tract asymptomatically resulting in faecal shedding (Schautteet and Vanrompay 2011, Hoffmann et al. 2015). This chlamydial species is of particular concern since many strains harbour a tetracycline resistance gene, which is likely transmitted within *C. suis*, and potentially to other chlamydial species, through homologous recombination (Lenart et al. 2001, Dugan et al. 2004, 2007, Suchland et al. 2009, Joseph et al. 2016, Seth-Smith et al. 2017b). *C. suis* has been isolated from farmers and slaughterhouse workers but no disease resulted from these zoonotic infections and none of the isolates were resistant to tetracycline (De Puysseleyr et al. 2014a,b, 2017, Kieckens et al. 2018). There is a single report of *C. suis* being detected in the eyes of trachoma patients from Nepal (Dean et al. 2013).

### Chlamydia sp. in pets

Apart from pet birds, guinea pigs and cats are known carriers of chlamydial species. *C. caviae* is present in the eyes and rectum of clinically healthy guinea pigs with prevalences <10% (2.7% in Switzerland and 8.9% in the Netherlands), but may induce conjunctivitis, pneumonia, and abortion in these hosts (Ciuria et al. 2021). This pathogen recently received attention as a cause of severe atypical pneumonia cases in humans (Ramakers et al. 2017). Before the emergence of these severe pneumonia cases in the Netherlands, *C. caviae* was known to cause conjunctivitis in guinea pig owners after close contact (Lutz-Wohlgroth et al. 2006).

*C. felis* is widespread and endemic in both, household cats and stray cats, causing conjunctivitis (Bressan et al. 2021). Its zoonotic potential is considered low with six cases of follicular conjunctivitis in human patients reported between 1969 and 2017 (Ostler et al. 1969, Darougar et al. 1978, Lietman et al. 1998, Hartley et al. 2001, Bomhard et al. 2003, Sykes 2005, Wons et al. 2017). A less common clinical picture of chronic follicular conjunctivitis with three cases, all related to cat contact, were recorded in the Netherlands between 2017 and 2022 (Hughes et al. 2024).

### *Chlamydia* sp. in exotic animals

The human respiratory pathogen *C. pneumoniae*, responsible for 7% of human CAP cases (Merida Vieyra et al. 2023), possesses one of the broadest host ranges of all known *Chlamydia* species, which includes mammals such as horses, marsupials (e.g. koalas) but also reptiles and amphibians (Sachse and Borel 2020). Evolutionary data indicate that human strains were zoonotically acquired (Roulis et al. 2013), however, no current zoonotic infections are published.

In recent years, reptile-specific chlamydial species have been identified, namely *C. poikilotherma, Candidatus* C. serpentis, and *Candidatus* C. corallus in snakes (Taylor-Brown et al. 2017, Staub et al. 2018), and *C. crocodili* as well as new *Candidatus* species in crocodiles (Chaiwattanarungruengpaisan et al. 2021, 2024). Their host spectrum as well as zoonotic potential is currently unknown and should be investigated further.

### Potential sources of zoonotic transmission

Zoonotic transmission has not only been associated with direct or indirect contact to farm animals and pets (e.g. contaminated bedding, pastures, instruments, and handling contaminated clothes). Wild animals and the environment are gaining importance as reservoirs for zoonotic chlamydial infections (Burnard and Polkinghorne 2016). In Sweden, psittacosis cases have recently been associated with wild birds and bird feeders in winter but without direct contact to domestic birds (Herrmann et al. 2024). Similarly, avian *C. abortus* cases involving 10 human infections and one mortality case also reported no animal contact, and remained without obvious source of infection (Raven et al. 2024). Moreover, there are human pneumonia cases of *C. caviae* that could not be linked to direct guinea pig contact (van Grootveld et al. 2018). These cases indicate a survival of chlamydiae in the environment. *Chlamydia psittaci* is well-known for its aerosolization capabilities as well as survival in dust, but more recently, other chlamydial species such as *C. suis* could be detected from dust in pig farm environments (Unterweger et al. 2024).

## The role of the plasmid in chlamydiae

### Occurrence and basic structure of the native chlamydial plasmid

In the early 1980s, researchers identified plasmid-like DNA in *C. trachomatis*. This double stranded circular DNA plasmid, ~7.5 kb in size, was subsequently found to be highly conserved across different *Chlamydia* species and strains, suggesting a pivotal role for both survival and virulence (Szabo et al. 2020).

**Figure 1.**
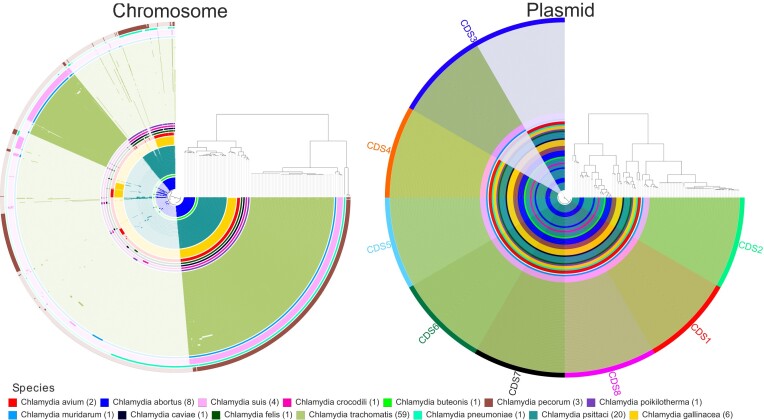
Pangenome analysis of 109 *Chlamydia* spp. genomes. All complete genomes from GenBank carrying a plasmid were included. Separate analysis was done for the chromosomal genes and the plasmid genes. The pangenomic analysis was done following the workflow pangenomic of Anvi'o 8 using Diamond and the default parameters for clustering the genes. The gene clusters are organized according to their distribution across the genome, with co-occurring genes shown closer together. The layers are colour coded per species and represent individual genomes organized by their phylogenomic relationships based on ANI.

In detail, the chlamydial plasmid exhibits several distinct properties: it possesses eight genes or coding sequences (CDS) with a copy number 4–10 times that of the chromosome (Thomas et al. 1997, Pickett et al. 2005). A detailed map of the plasmid from *C. trachomatis* and its coevolution with the chromosome has been described (Seth-Smith et al. 2009). A recent study further compared the plasmids of 10 recognized chlamydial species and identified three distinct plasmid lineages of which the first comprised *C. pecorum* and *C. pneumoniae*, the second *C. trachomatis, C. suis*, and *C. muridarum*, and the third all remaining species (*C. psittaci, C. felis, C. caviae, C. avium*, and *C. gallinaceae*) (Szabo et al. 2020). These plasmid lineages coincided with the corresponding genotypes that were based on the *ompA* gene that encodes for the major outer membrane protein (Szabo et al. 2020). Here, we included the remaining four recognised species (*C. abortus, C. buteonis, C. crocodili*, and *C. poikilotherma*) and observed a similar but not identical stratification of the whole chromosome into four major clusters (Fig. 1). Specifically, the four additional species fell into the largest clade, which shares a significant core-genome [average nucleotide identity (ANI) >77.8%]. *C. trachomatis, C. suis*, and *C. muridarum* belong to a distinct second clade (ANI > 80.8%), followed by two mono-specific clades for *C. pneumoniae* and *C. pecorum* (Fig. 1). The plasmid sequences were conserved and only CDS3 showed significant variation within the clades with *C. trachomatis, C. muridarum*, and *C. suis* diverging from the other clades (Fig. 1).

The individual properties of the chlamydial plasmid encoded proteins have also been studied and they are involved in DNA replication, plasmid maintenance, and modulation of host cell functions (Zhong 2017). The plasmid encodes a strong promoter driving the transcription of short antisense RNAs. There are additional promoters and a complex transcription profile with temporal controls as well as some CDS with transcriptional start points (Ricci et al. 1993, 1995).

The plasmid enhances pathogenicity and infection efficiency. For example, the plasmid-protein Pgp3, encoded by CDS 5, is known to be involved in immune modulation and enhancing ability of the bacterium to invade specific host cells (Huo et al. 2020). These properties underscore the role of the plasmid in fine-tuning the adaptability and pathogenicity of *Chlamydia*.

### Plasmid-free *Chlamydia* strains

Notably, the plasmid is not essential for the existence of the bacterium, as plasmid-free *Chlamydia* strains occur naturally. However, only three such ‘live’ clinical isolates of *C. trachomatis* have been described (Peterson et al. 1990). In *C. trachomatis*, the loss of the plasmid *in vivo* is likely associated with a loss of virulence, which amounts to a loss of fitness and ultimately an extinction event for that specific plasmid. This is because such strains fail to propagate in the human population, consistent with the notion of loss of virulence. However, plasmid loss can occur spontaneously *in vitro* (Matsumoto et al. 1998). Furthermore, the plasmid can be ‘cured’ chemically (O’Connell and Nicks 2006, Skilton et al. 2018). In both *C. trachomatis* and *C muridarum*, loss of the plasmid results in a change of inclusion phenotype and loss of the ability to synthesize glycogen within the inclusion. There is phylogenetic evidence, based on SNP analyses, that the plasmid can be transferred naturally between chlamydiae from the rare detection of recombination between plasmids in *C. trachomatis* isolates (Harris et al. 2012). In a comprehensive follow-up study of chlamydial genomes, plasmid replacement between lineages confirmed the natural transfer of the plasmid (Hadfield et al. 2017). This can only have occurred through mixed infection and plasmid exchange. By contrast, there are entire clusters within other chlamydial species that are stably plasmid-free such as the human *C. pneumoniae* isolates (Table 1) and all known ruminant *C. abortus* strains as opposed to the avian strains that all carry a plasmid (Seth-Smith et al. 2017a, Longbottom et al. 2021, Zaręba-Marchewka et al. 2021).

In *C. pecorum*, while the plasmid is frequently found in strains originating from different hosts, plasmid-free strains have been characterized and may be common among bovine strains (Islam et al. 2019, Jelocnik et al. 2023, Hagenbuch et al. 2024). It has been speculated that the plasmid plays a role as a virulence factor in koala strains, but this could not be confirmed in a study comparing different strains (Fernandez et al. 2023). Interestingly, a study of abortigenic ovine *C. pecorum* strains found a distinct link between virulence and a 34-bp deletion in the nonessential CDS 1 of the plasmid (Jelocnik et al. 2023).

In contrast, almost all known strains of *C. suis* are plasmid-bearing except for one strain. The significance of the *C. suis* plasmid as a virulence factor is currently unclear (Joseph et al. 2016, Seth-Smith et al. 2017b).

### The significance of the chlamydial plasmid in diagnostics and vaccine development

Because the plasmid is highly conserved in *C. trachomatis* and present in nearly all clinical isolates, it serves as an excellent target for diagnostic tests, helping to identify infections. However, in 2006, a genital tract strain of *C. trachomatis* emerged in Sweden carrying a deletion that escaped detection by two of the main commercial tests (Ripa and Nilsson 2007). This became an exemplifier of selection by diagnostic failure and subsequent lack of treatment. As a result, dual target testing became the norm in screenings for STI. In veterinary medicine, most tests continue to use only one target gene, which, for chlamydiae, tend to be located on the chromosome such as the gene encoding for the major outer membrane protein (*ompA*) and the 23S ribosomal RNA sequence (Pantchev et al. 2009, 2010). One of the reasons why the plasmid has not been considered as the primary diagnostic target is the absence of plasmid in many veterinary chlamydiae, especially in *C. abortus*. However, the Pgp3 protein of *C. trachomatis* and *C. psittaci* has been used as a target for serological testing of animal and human infections (Donati et al. 2009).

The plasmid’s nonessential nature also makes it an attractive candidate for developing plasmid-free *C. trachomatis* strains for use in live attenuated vaccines (Kari et al. 2011), but not in veterinary chlamydiae given its uncertain role as a virulence factor. By understanding the plasmid’s structure and function in each individual chlamydial species, scientists are exploring ways to disrupt its role in infection, paving the way for novel treatment strategies. This research highlights the plasmid’s significance not only in basic microbiology but also in advancing clinical interventions against chlamydial diseases.

### The native plasmid as a tool for genetic modification

The obligate intracellular nature of the *Chlamydiaceae* family has impeded efforts to understand the biology of these complex bacteria. However, the stable and conserved nature of the chlamydial plasmid has facilitated the development of tools for genetic manipulation of the *Chlamydiaceae* family (Wang et al. 2011). This has opened new avenues for studying chlamydial genetics including the molecular mechanisms underlying chlamydial infections for example virulence, host interactions, and immune evasion strategies (O’Neill et al. 2020, Banerjee and Nelson 2021). In the next chapter, we will discuss currently available tools for all recognized *Chlamydia* species as well as important findings that could be crucial for future gene modification trials.

## Transformation of human, zoonotic, and other veterinary *Chlamydia* species

Since Wang et al. (2011) demonstrated stable transformation of *C. trachomatis* using a species-specific shuttle vector, various strategies for editing the chlamydial genome or mRNA expression have been established by different research groups. They include protein expression systems under promoter control (Bauler and Hackstadt 2014), TargeTron (Johnson and Fisher 2013, Weber et al. 2016), fluorescence-reported allelic exchange mutagenesis (FRAEM) (Mueller et al. 2016), floxed-cassette allelic exchange mutagenesis (FLAEM) (Keb et al. 2018), CRISPRi (Ouellette 2018), sRNAs (Wang et al. 2022), and transposon mutagenesis (LaBrie et al. 2019, Wang et al. 2019, O’Neill et al. 2021).

While these novel techniques enable the characterization of chlamydial virulence factors primarily in *C. trachomatis*, there are fewer tools available for other *Chlamydia* spp. Table 1 lists all currently known transformation systems developed for all 14 recognised chlamydial species. Specifically, TargeTron was successfully used for *C. caviae* (Filcek et al. 2019), allelic exchange vectors for *C. suis* and *C. psittaci* (Binet and Maurelli 2009, Marti et al. 2023), and a protein expression system under a tetracycline promoter-control system was developed for *C. psittaci* (Shima et al. 2020). However, by far the most commonly used transformation system for chlamydial species other than *C. trachomatis* are shuttle vector systems, which have been developed for *C. caviae, C. felis, C. muridarum, C. peccorum, C. pneumoniae, C. psittaci*, and *C. suis* (Wang et al. 2014, Shima et al. 2018, 2020, Marti et al. 2023, Faessler et al. 2024).

### The basics of chlamydial transformation using shuttle vectors

Chlamydial plasmid shuttle vectors typically utilize the complete native plasmid, which are introduced into the *Chlamydia* species of interest by calcium chloride (CaCl_2_)-mediated transformation leading to stable transformants both in the presence and absence of selection. *C. trachomatis* plasmid shuttle vector pGFP::SW2 was the first shuttle vector described in literature (Wang et al. 2011). It contains all eight CDS of the plasmid pSW2 derived from *C. trachomatis* strain SW2 as a backbone, of which the first was interrupted by an inserted *bla* gene, an *Escherichia coli* origin of replication (*ori*) as well as a *cat* gene fused with red-shifted green fluorescent protein gene (RSGFP) placed under a *Neisseria meningitidis* promoter (nmP). In other plasmid shuttle vectors, the native plasmid is disrupted between CDS 1 and 2 (Bauler and Hackstadt 2014).

### Barriers of transformation

It has been suggested that plasmid shuttle vectors must be constructed with the same chlamydial backbone as the plasmids harboured by the same chlamydial species. In fact, Song et al. (2014) demonstrated that successful transformation of *C. trachomatis* and *C. muridarum* was observed only when the plasmid shuttle vector used for transformation possessed a compatible parental chlamydial plasmid backbone. In their study, *C. trachomatis* serovar A could not be transformed with a shuttle vector comprising an L2 plasmid backbone.

One of the factors identified as conferring compatibility was the CDS 2 region of the native plasmid (Song et al. 2013, Wang et al. 2014). However, the exact dynamics are not entirely clear, as some chlamydial shuttle vectors have crossed biovar, genotype, and even species borders. For example, different *C. trachomatis* serovar E shuttle vectors were successfully transformed into *C. trachomatis* serovars A, D, and L2 (Wang et al. 2011, Ding et al. 2013, O’Neill et al. 2018). Moreover, *C. pneumoniae* plasmid shuttle vector pRSGFPCAT-Cpn, derived from horse *C. pneumoniae* strain N16, could not only be transformed into koala strain LPCoLN and naturally plasmid-free human strains CV-6 (cardiovascular isolate) and IOL-207 (CAP-associated) but was also stably introduced into three different *C. felis* strains without recombination of the plasmid. Even though the *C. pneumoniae* genome is genetically closer to *C. pecorum* than to *C. felis*, the *C. pneumoniae* shuttle vector could not be introduced into *C. pecorum* (Fig. 1) (Shima et al. 2018). These findings indicate that the similarity of genomic and plasmid sequences does not entirely explain the barriers of transformation.

One major drawback for all chlamydial species is a very low transformation efficiency independent of the transformation protocol (O’Neill et al. 2021, Marti et al. 2023). Factors such as CaCl_2_ concentration, selection antibiotics, and strains of choice but also the exact infection protocol play a significant role in increasing the rate of transformation (Marti et al. 2023). For example, a recent study in *C. caviae* showed that, while the protocol established for *C. trachomatis, C. psittaci*, and *C. pneumoniae* (Wang et al. 2011, Shima et al. 2018, 2020) was successful, the protocol optimised for *C. suis* (Marti et al. 2023) was not (Faessler et al. 2024). These protocols comprise different CaCl_2_ concentrations (50 mM versus 100 mM), vector/*Chlamydia* coincubation times (30 min versus 1 h), and additional coincubation with trypsinized cells (20 min versus none). The detailed protocols are published in protocols.io (https://dx.doi.org/10.17504/protocols.io.kxygxy53wl8j/v1). Interestingly, transformation attempts with *C. abortus* remained unsuccessful with either protocol (Faessler et al. 2024). The *C. abortus* shuttle vector derived from the avian strain 15–70d24 (Zaręba-Marchewka et al. 2019) and transformation was attempted for both, a ruminant strain and 15–70d24. These results indicate that, while there may be true barriers of transformation between the different species, not all transformation protocols work for all species, and adaptations may be necessary to improve the transformation efficiency for individual species and strains.

### Plasmid sequence-independent transformation

Vectors without the plasmid backbone sequence of any *Chlamydia* species have been transformed into both *C. trachomatis* and *C. suis* (Binet and Maurelli 2009, Garvin et al. 2021, Marti et al. 2023). All vectors contained sequences that were homologous with the chromosomal target gene, which enabled allele replacement. Interestingly, an allele replacement vector containing the genomic *C. suis trpBA* operon was successfully integrated into *C. trachomatis* but not *C. muridarum*, although its phylogenetic relationship to *C. suis* is closer (Fig. 1) (Marti et al. 2023). Vectors with only the chlamydial plasmid origin of replication have also been successfully introduced (Fields et al. 2022). These minimal replicon vectors could be applied as gene deletion tools for studies as they do not replace the native plasmid, as is common for vectors containing the whole plasmid, and are unstable in the absence of antibiotic selection.

Finally, TargeTron shuttle vectors such as pDFTT3, pDFTT3-CAT, and pACT as well as transposon shuttle vectors such as pCMA and pCMC5M encode only chlamydial promoter regions but not chlamydial plasmid backbone sequences (Johnson and Fisher 2013, Weber et al. 2016, Filcek et al. 2019, LaBrie et al. 2019, Wang et al. 2019, DeBoer et al. 2023, Karanovic et al. 2023). Mostly used for *C. trachomatis* and *C. muridarum*, this has also been successfully applied for *C. caviae* (Table 1) and is therefore a feasible alternative to shuttle vector transformation for other zoonotic and veterinary chlamydial species.

## Outlook/conclusion

Zoonotic infections due to chlamydiae in humans, such as CAP or miscarriage, often remain undiagnosed and underreported. The identification of new chlamydial species in various hosts as well as examples of new disease manifestations and infection sources demonstrate that diagnostic investigations into CAP or miscarriage must expand. Specifically, diagnostic tests should go beyond *C. psittaci* and *C. pneumoniae* for CAP, or *C. abortus* for miscarriage, either by a broader screening targeting the entire *Chlamydiaceae* family, or by expanded species-specific testing. Moreover, extended patient histories concerning the direct or indirect contact to wildlife, pets, and livestock, are crucial to discover new reservoirs and potential sources of transmission. The conserved plasmid of the chlamydiae could serve as an excellent screening method, particularly if it is combined as a dual approach with a chromosomal target to avoid overlooking the presence of plasmid-free strains, particularly among *C. pneumoniae, C. abortus*, and *C. pecorum*.

The plasmid could further serve as a fascinating area of study given its unclear status as a virulence factor, which appears to be species- and possibly even host species-specific. In particular, *C. abortus* with the plasmid-free ruminant and plasmid-carrying avian strains could serve as model organism to investigate whether tissue tropism, host specificity as well as virulence are tied to the presence of a plasmid. Furthermore, the plasmid remains an intriguing target for vaccine development and has been indispensable for the development of gene modification approaches.

Finally, the variety of available tools for gene modification among the *Chlamydia* has considerably increased and could be successfully applied to 8 of the 14 recognised *Chlamydiaceae* species. However, despite efforts to optimise existing transformation protocols, not all chlamydial species and strains could be genetically modified and require further attention. The development of such tools could help to unravel the versality of the *Chlamydiaceae* by gaining a better understanding of known and identifying new virulence factors that are unique to this bacterial family.
